# High proportion of young children are vitamin D sufficient after expansion of Sweden’s mandatory fortification but dairy products also contribute to a high climate impact

**DOI:** 10.1186/s12937-026-01318-6

**Published:** 2026-04-01

**Authors:** André Hesselink, Anna Winkvist, Anna Karin Lindroos, Lotta Moraeus, Helena Bjermo, Sanna Lignell, Linnea Bärebring, Elinor Hallström, Hanna Augustin

**Affiliations:** 1 Department of Internal Medicine and Clinical Nutrition, Institute of Medicine, Sahlgrenska Academy, University of Gothenburg, Box 459, Gothenburg, 405 30 Sweden; 2 Department of Food Studies, Nutrition and Dietetics, Uppsala University, Uppsala, Sweden; 3 Department of Risk and Benefit Assessment, Swedish Food Agency, Uppsala, Sweden; 4 Unit for Sustainable Food Consumption, Department of Food Research and Innovation, RISE Research Institutes of Sweden, Lund, Sweden

**Keywords:** Micronutrients, Dietary intake, 25-hydroxyvitamin D, Greenhouse gas emissions, Fortification policy

## Abstract

**Background:**

Sweden expanded its mandatory vitamin D fortification program in 2018 addressing high rates of insufficient vitamin D status and low intake. This study aimed to assess vitamin D status among young children post-expansion, evaluate its determinants, and identify main vitamin D sources and their climate impact.

**Methods:**

Dietary intake among 18-month-olds (18mo, *n* = 1074) and 4-year-olds (4yo, *n* = 746) were obtained from the Swedish national survey Riksmaten Young Children 2021-24 based on two non-consecutive 24-hour food records. Intake of vitamin D from drops (µg/d) was also recorded. Individual vitamin D intakes were adjusted using the Multiple Source Method to reflect habitual intake. Blood concentrations of 25-hydroxyvitamin D (25OHD) were provided for a subset (18mo, *n* = 281; 4yo, *n* = 270). Climate impact was expressed as greenhouse gas emissions (GHGEs) in carbon dioxide equivalents (CO₂ eq), using life cycle assessment data from the Research Institutes of Sweden. Associations between 25OHD and plausible determinants were analyzed with linear regression.

**Results:**

16% of 18mo and 61% of 4yo had total vitamin D intakes (including diet and drops) below the average requirement, 7.5 µg/d. None were deficient (25OHD < 30 nmol/l), and 7% of 18mo and 4% of 4yo were insufficient (< 50 nmol/l). 25OHD was positively associated with dietary vitamin D intake and vitamin D from drops, and inversely associated with blood sampling during winter and parents’ birth country (≥ 1 parent born outside Nordics). Main vitamin D sources for 18mo were vitamin D drops and fortified foods: dairy, porridge and cereal drinks; and for 4yo, fortified dairy and fat spreads. Dairy was a large contributor to diet-related GHGEs (18mo: 10%, 4yo: 16%), whereas fortified fat spreads and plant-based dairy alternatives were effective sources of vitamin D, providing the highest vitamin D concentrations per kg CO₂ eq.

**Conclusions:**

A high proportion of young children in Sweden are vitamin D sufficient, with dietary vitamin D intake, vitamin D drops, and blood sampling season being major determinants. Fortified dairy is an important dietary source of vitamin D, but a shift to fortified plant-based alternatives would reduce diet-related climate impact and should be explored in future research.

**Supplementary Information:**

The online version contains supplementary material available at 10.1186/s12937-026-01318-6.

## Background

Vitamin D is a key nutrient for proper bone mineralization and skeletal development with deficient vitamin D status associated with rickets in children and osteomalacia in adults [1, 2]. Biological mechanisms and observational data also suggest health benefits of vitamin D related to cardiovascular disease, blood glucose regulation, immune response, cancer and all-cause mortality [3]. The human body can synthesize vitamin D from ultraviolet B (UVB) light via sun exposure. However, this UVB radiation is not sufficient during winter months in the Nordics and at other latitudes above 51 degrees [4]. When endogenous production is insufficient, vitamin D must be obtained from the diet or supplements. Major food sources include fatty fish (e.g., salmon, trout, mackerel, herring), egg yolk and fortified products [5]. In Sweden, the average requirement (AR) for vitamin D is 7.5 µg/d and the recommended intake is 10 µg/d, as established in the Nordic Nutrition Recommendations 2023 (NNR 2023) [5]. All children up to 2 years of age are recommended to receive oral supplementation with vitamin D drops providing 10 µg/d [6].

Vitamin D status is measured by circulating blood concentrations of 25-hydroxyvitamin D (25OHD). While there is no global consensus, concentrations below 30 nmol/l are often considered vitamin D deficient and 30–50 nmol/l insufficient [1, 2, 5]. Studies in Sweden among 10–18 year-olds have shown prevalences of deficiency ranging from 4 to 16% and insufficiency from 14 to 62% (*n* = 206, 2014 [7]; *n* = 1100, 2016-17 [8]). Reported dietary intakes of vitamin D have also been low with 60–90% of children aged 4–12 years (*n* = 590, 2003 [9]) and adolescents aged 14–18 years (*n* = 3099, 2016-17 [8]) having intakes below the AR.

Milk (≤ 1.5% fat), margarine and other fat spreads have been fortified in Sweden with vitamin D since 2007. This mandatory fortification was expanded in 2018 to address the poor vitamin D status in the population, increasing fortification levels and the number of products required to be fortified [10]. New products included all milk and yoghurt (≤ 3% fat) and plant-based milk and yoghurt alternatives. Most porridge and *välling* (a cereal drink popular in Sweden) products intended for young children are also fortified with vitamin D.

Major dietary sources of vitamin D for young children (4–12 years) in Sweden include dairy, fat spreads, fish, children’s porridge and cereal drinks, red meat and poultry [9, 11]. Among these sources, animal-based products (e.g., red meat and dairy) contribute significant amounts of greenhouse gas emissions (GHGEs) [12, 13] and their consumption is often limited in reference diets promoting climate sustainability [14]. A diet optimization study using linear programming based on the diets of Swedish adolescents showed a high reliance on fortified dairy products to reach adequate vitamin D intake and remain within per capita planetary boundaries for climate change [15]. A similar study looking at adults in the Netherlands concluded that achieving adequate intake and limiting GHGEs from the diet to an acceptable level was only possible by expanding vitamin D fortification to other products such as bread, milk and oils [16].

Riksmaten Young Children 2021-24 is the first large-scale, national dietary survey to be completed after the expansion of Sweden’s mandatory vitamin D fortification policy. The food and nutrient data collected (18-month-olds, *n* = 1074; 4-year-olds, *n* = 746) provides the first opportunity to evaluate the effectiveness of this fortification policy expansion in these age groups, from both a human health and climate sustainability perspective. New insights from current vitamin D intake and status, main food sources and climate impact can help shape future fortification policy and dietary guidelines. Thus, the aims of this study were to (1) assess vitamin D intake and status among young children in Sweden within the context of fortification, (2) explore factors associated with vitamin D status, and (3) identify main dietary sources and their climate impacts.

## Methods

### Study design and population

The national dietary survey Riksmaten Young Children was conducted by the Swedish Food Agency from 2021 to 2024. Children 9 months, 18 months and 4 years old across Sweden were randomly selected from government population registries. For this study, only 18-month-olds (18mo, *n* = 1078) and 4-year-olds (4yo, *n* = 750) were included in the analyses with final participation rates of 12% and 10%, respectively. Roughly 28% of participants came from Middle Sweden, 23% South/Southeast, 20% Stockholm (capital) region, 18% West and 10% North. Blood samples were collected for a sub-sample (18mo: *n* = 294, 4yo: *n* = 273), including only participants living within 50 km of one of seven Environmental and Occupational Medicine (EOM) clinics located from Umeå in the north to Lund in the south of Sweden. No blood samples were collected from the 9-month-olds, and that age group was thus not included in this study. Among participants asked to provide biological samples, 51% agreed. A more detailed description of Riksmaten Young Children 2021-24 has been published previously [17]. Informed written consent was obtained from parents or legal guardians for all participants. Ethical approval for Riksmaten Young Children was obtained from the Swedish Ethical Review Authority (registration nos. 2020–05293, 2021–03591).

### Dietary assessment and background variables

Dietary intakes were assessed via a web-based, 24-hour food record method, RiksmatenFlex [18], recorded on two non-consecutive days (1 weekday, 1 weekend day) by participants’ parents. The parents were instructed to start the record on a weekend, and then a second weekday was randomly assigned. If applicable, daycare staff filled in paper records, which were then registered in the web-based system by the parents. A food record was considered complete if (1) both days were registered and (2) neither day was less than 100 kJ in reported energy intake [17]. Sensitivity analyses of possible outliers performed by the original survey investigators concluded that all records meeting these two requirements could be included in follow-on analyses [19]. RiksmatenFlex, used as a 24-hour recall, has been validated against in-person 24-hour recalls and objective biomarkers in adolescents, and has shown comparable results for energy and intakes of fruit, vegetables, whole grain wheat and rye [18]. RiksmatenFlex is currently being validated in young children and intake of vitamin D agrees well with the intake obtained by 24-hour recall interviews (Lindroos AK, personal communication).

A list of 1218 foods, beverages and composite dishes (collectively, foods) was available to choose from the Swedish Food Agency’s food composition database, version Riksmaten young children 2021-24, 20240201. The food list included items often consumed by toddlers and young children, and food portions were adjusted to accommodate smaller portion sizes (e.g., teaspoons) and consistencies (e.g., purées). Vitamin D supplements in the form of drops could also be registered (1 drop = 2 µg vitamin D), and participants were reminded to do so, if applicable. Responses were converted to edible weights or volume and linked to the food composition database for automatic assessment of nutrient intakes [17]. Riksmaten Young Children also collected information covering child and parents’ backgrounds, child’s height and weight, socio-demographic information, supplement use and food habits via a self-administered questionnaire. This questionnaire has been published elsewhere [19].

Vitamin D drops registered in the food record are included in the calculation of participants’ total vitamin D intake. Usage of other supplements containing vitamin D was captured via the questionnaire but not quantifiable, parents answering yes or no with the option to specify further in free text. Since individual dietary assessments covered only two days and certain vitamin D-rich foods can be consumed infrequently, vitamin D intakes were adjusted using the Multiple Source Method (MSM) [20] to reflect long-term, habitual intake of vitamin D. In short, an estimated probability of vitamin D intake from the diet on any given day is multiplied by an estimated daily amount to arrive at long-term adjusted intake for each individual. The MSM-adjusted values for total vitamin D intakes (from the diet and vitamin D drops) were used to determine the proportion of individuals in this sample population below the AR. The AR for vitamin D was set at 7.5 µg/d as established in the NNR 2023 [5]. For the linear regression analyses described below, two separate intake variables were constructed to distinguish the effect estimates between dietary vitamin D and vitamin D from drops.

For analysis purposes, foods consumed were categorized into 33 categories as defined in the Riksmaten Young Children survey. A detailed list can be found in the supplementary materials (see Additional file 1).

### Blood sampling and vitamin D analysis

Blood samples were collected by venous puncture at one of the seven EOM clinics. Sampling was performed by certified personnel and local anesthetic applied prior to sampling. Samples were centrifuged (2200 g, 10 min) after waiting ≥ 30 min (max 5 h), and plasma pipetted into aliquots and stored at -80 °C at the EOMs. Frozen samples were later transported to the Swedish Food Agency and stored at -80 °C until analysis. Plasma concentrations of 25OHD_2_, 25OHD_3_ and 3-epi-25-OH-D_3_ were assessed by VITAS Analytical Services, Oslo, Norway (18mo, *n* = 281; 4yo, *n* = 270) using high-performance liquid chromatography coupled with atmospheric pressure chemical ionization tandem mass spectrometry (HPLC-APCI-MS/MS). The analytical platform was certified by the Vitamin D External Quality Assessment Scheme (DEQAS) [19]. The intra-assay coefficient of variation for 25OHD₃ ranged from 5.3% to 7.2% across three concentration levels (60, 105 and 216 nmol/l). Inter-assay variation could not be assessed as analyses were the first performed on this platform (analysis date: April 2024).

Vitamin D status was reported as total 25OHD, calculated as the sum of 25OHD_2_ and 25OHD_3_. 3-epi-25-OH-D_3_ was not included. Limits of quantification (LOQ) for 25OHD_2_ and 25OHD_3_ were 2 nmol/l and 5 nmol/l, respectively. 25OHD_3_ was detectable in all samples. Values for 25OHD_2_ below the LOQ were set to zero (*n* = 321, 58% of samples). Plasma concentrations of total 25OHD below 30 nmol/l were deemed deficient and between 30 and 50 nmol/l as at risk for insufficient status [21]. Blood sampling season was dichotomized to April to September (“summer”) and October to March (“winter”) to represent periods of high and low sun exposure, respectively [22].

### Climate impact estimates of foods consumed

Climate impact estimates of foods in the survey are based on life cycle assessment (LCA) data available from the Research Institutes of Sweden’s (RISE) Food and Climate Database, version 2.03 [23]. Riksmaten survey foods were matched to climate data in the RISE database, and in the case of composite dishes, matching was based on pre-defined recipes. LCA estimates were not available for supplements, including vitamin D drops. Climate impact estimates at food category levels (33 categories defined in Riksmaten Young Children) were derived using weighted averages of the individual estimates based on actual consumption from food records.

Climate impact, i.e., diet-related GHGEs, include all relevant greenhouse gases (e.g., methane, nitrous oxide, carbon dioxide) from primary production up to and including industry gate. Transportation to Sweden for imported foods is included, but packaging and home preparation are excluded for all foods. Calculations assume a 100-year global warming potential for GHGEs and estimates are measured in kg of CO_2_-equivalents (CO_2_ eq) per kg of edible amount [23].

### Statistical analysis

All statistical analyses were performed in RStudio 2023.12.0 running on R version 4.3.3, Linux Ubuntu 22.04.4. *P*-value < 0.05 was the threshold for statistical significance.

Determinants of vitamin D status were selected based on current literature [24] and biological plausibility together with directed acyclic graphs (DAGs) for potential causal effects and confounding (see supplementary materials, Additional file 1). Variables were checked for normality where relevant (histograms, Shapiro-Wilk test) and data imbalances via frequency tables. For the linear regression analyses, only dietary vitamin D intake required adjustment, using the MSM method (described above) to reflect habitual intake.

Final variables included dietary vitamin D intake (µg/d, MSM-adjusted), vitamin D intake from drops (µg/d), age group, blood sampling season (0: summer, 1: winter), parents’ birth country (0: both parents born in Nordics (Sweden, Norway, Denmark, Finland and Iceland), 1: at least one parent born outside Nordics), household education (0: neither parent with education > 12 years, 1: at least one parent > 12 years) and supplements containing vitamin D other than drops (0: No, 1: Yes). The final variable, supplements containing vitamin D other than drops, was only included in the regression for 4yo since so few children among 18mo were given vitamin D supplements other than drops. Determinants were checked for multicollinearity, and age group showed correlation coefficients of -0.64 and + 0.34 with vitamin D intake from drops and other supplements, respectively.

Linear associations between the determinant variables and 25OHD concentration were assessed using multivariable regression models, stratified by age group and blood sampling season. Regression residuals were evaluated for normality and homoscedasticity. Potential interaction effects were investigated through regression analyses and data visualizations. Interaction terms were tested in regression models between habitual dietary vitamin D intake and blood sampling season, parents’ birth country and household education. No interaction terms were significant (data not shown).

## Results

The proportions of girls and boys were similar across age groups with 48% girls among 18mo and 49% among 4yo (Table 1). More than 80% of participants in both age groups had both parents born in a Nordic country. Similarly, approximately 80% lived in either the middle or southern part of Sweden and a high proportion of households (18mo: 86%, 4yo: 87%) with at least one parent with more than 12 years education. A more detailed breakdown of parents’ birth countries is available in the supplementary materials (see Additional File 1).

**Table 1:** Background characteristics of Riksmaten Young Children participants (*n* = 1820)

	18-month-olds	4-year-olds
Number of participants	1074	746
Sex, female, *n* (%)	512 (48)	362 (49)
Age, years, mean (SD)	1.5 (0.1)	4.1 (0.1)
IOTF weight status, *n* (%): ^a^		
Underweight	-	89 (12)
Normal	-	518 (69)
Overweight	-	49 (7)
Obese	-	12 (2)
Parents’ birth country, n (%): ^b^		
Both parents, Nordics	876 (82)	607 (81)
One parent, outside Nordics	129 (12)	99 (13)
Both parents, outside Nordics	69 (6)	44 (6)
Geographic location in Sweden, n (%): ^c^		
North, latitudes 59º- 69º	178 (17)	129 (17)
Mid, 58º- 61º	446 (41)	326 (44)
South, 55º- 59º	454 (42)	291 (39)
Household education (> 12 years), n (%)	925 (86)	647 (87)

^a^ Weight status based on age-specific (in months) BMI cut-offs from 24 months defined by the IOTF [25]. For reference, BMI cut-offs for age 4 years are as follows. Underweight (thinness, grade 1): boys < 14.51, girls < 14.30; Overweight: boys ≥ 17.53, girls ≥ 17.36; Obese: boys ≥ 19.23, girls ≥ 19.16

^b^ Nordics = Sweden, Norway, Denmark, Finland, Iceland

^c^ Geographic location as defined by European Union’s Nomenclature of territorial units for statistics (NUTS) classification system, level 1 [26]. Latitudes approximated using Google Maps [27]

*Abbreviations: BMI* Body mass index, *IOTF* International Obesity Taskforce, *SD* Standard deviation

### Total vitamin D intake and status

Means for total vitamin D intake were 12.3 µg/day (SD: 4.4) and 7.1 µg/day (SD: 3.1) for 18mo and 4yo, respectively, with 16% of 18mo and 61% of 4yo below the AR of 7.5 µg/day [5] (Table 2). Mean plasma concentrations of 25OHD were 73.1 (SD: 16.2) and 69.3 nmol/l (SD: 13.2), respectively, with 7.1% of 18mo and 4.4% of 4yo below 50 nmol/l [21]. No children in either age group had 25OHD concentrations below 30 nmol/l. Blood was sampled from October to March for 59% of 18mo and 45% of 4yo. Stratification by month of blood sampling showed very few 18mo sampled during June to September, whereas 4yo were evenly spread across all months of the year. This detail can be found in the supplementary materials (see Additional file 1).

**Table 2:** Vitamin D intake and status of Riksmaten Young Children participants

	18-month-olds	4-year-olds
Participants with completed food records	1074	746
Energy, kcal/d (SD)	1160 (283)	1428 (328)
Vitamin D intake, mean (SD): ^a^		
Total intake (diet and vitamin D drops), µg/d	12.3 (4.4)	7.1 (3.1)
Dietary intake (excl. drops), µg/d	6.2 (2.5)	6.6 (2.4)
Vitamin D from fortified foods (% of total intake)	38%	67%
Using vitamin D drops, n (%)	1017 (95)	130 (17)
Using other supplements with vitamin D, n (%) ^b^	53 (5)	215 (29)
Proportion, % below AR (95% CI) ^a c^	16 (14, 18)	61 (58, 65)
Participants with plasma sample for 25OHD	281	270
Total 25OHD in nmol/l, mean (SD):	73.1 (16.2)	69.3 (13.2)
< 50 nmol/l, n (%)	20 (7.1)	12 (4.4)
< 30 nmol/l, n (%)	0 (0.0)	0 (0.0)
Blood sampling: winter, n (%) ^d^	167 (59)	121 (45)

^a^ Individual vitamin D intakes adjusted by the Multiple Source Method to reflect habitual intake

^b^ Does not include vitamin D drops

^c^ Total vitamin D intake used to determine proportion below the AR. The AR and RI for vitamin D in Sweden are 7.5 and 10 µg/d, respectively [5]

^d^ Blood sampling, winter: October-March

*Abbreviations:AR* Average requirement, *CI* Confidence interval, *25OHD* 25-hydroxyvitamin D, *RI* Recommended intake, *SD* Standard deviation

### Determinants of vitamin D status

Multivariable regression analyses (Tables 3 and 4), including all participants, showed positive associations between dietary vitamin D intake and 25OHD concentrations in both 18mo (β = 1.02, *p* = 0.004) and 4yo ( β = 1.22, *p* < 0.001). Vitamin D intake from drops was also positively associated with 25OHD in both age groups (18mo: β = 0.60, *p* = 0.006; 4yo: β = 0.86, *p* = 0.011). Blood sampling during winter (October to March) showed an inverse association with 25OHD (β=-5.38, *p* = 0.001), but only among 4yo. Having at least one parent born outside Nordics was associated with lower 25OHD among 18mo (β=-4.89, *p* = 0.039).

**Table 3:** Multivariable linear regression analysis (25OHD, nmol/l) stratified by blood sampling season, 18-month-olds

18-month-olds	Full year (*n* = 281)			Summer (*n* = 114)			Winter (*n* = 167)		
	β	SE	*p*-value	β	SE	*p*-value	β	SE	*p*-value
Vitamin D (µg/d):									
Dietary intake ^a^	1.02	0.35	0.004	1.83	0.57	0.002	0.58	0.44	0.194
Vitamin D drops	0.60	0.22	0.006	0.85	0.36	0.019	0.48	0.29	0.094
Blood sampling season ^b^	-2.73	1.92	0.157	---	---	---	---	---	---
Parents’ birth country ^c^	-4.89	2.35	0.039	-3.91	3.55	0.274	-5.34	3.20	0.097
Household education ^d^	-1.48	3.06	0.629	-1.60	4.28	0.709	-2.19	4.43	0.621

Adjusted R-squared: Full year = 0.062, summer = 0.113, winter = 0.021

^a^ Adjusted to reflect habitual consumption using the Multiple Source Method

^b^ Blood sampling season: summer (April-September) = 0, winter (October-March) = 1

^c^ Parents’ birth country: At least one parent born outside Nordics (Sweden, Norway, Denmark, Finland, Iceland) = 1, otherwise = 0

^d^ Household education: At least one parent with ≥ 12 years education = 1, otherwise = 0

*Abbreviations:25OHD* 25-hydroxyvitamin D, *SE* Standard error

When stratifying by season of blood sampling, the beta coefficients for dietary vitamin D intakes and vitamin D intake from drops, in relation to 25OHD, were higher during summer sampling than winter among 18mo, but no longer significant during winter. The opposite was true of 4yo with higher beta coefficients during winter, but only dietary vitamin D was significantly associated with 25OHD during summer. Furthermore, after stratification for season, parents’ birth country was no longer associated with 25OHD in either age group. Instead, use of supplements containing vitamin D other than drops was positively associated with 25OHD among 4yo during winter (β = 4.85, *p* = 0.026).

**Table 4:** Multivariable linear regression analysis (25OHD, nmol/l) stratified by blood sampling season, 4-year-olds

4-year-olds	Full year (*n* = 270)			Summer (*n* = 149)			Winter (*n* = 121)		
	β	SE	*p*-value	β	SE	*p*-value	β	SE	*p*-value
Vitamin D (µg/d):									
Dietary intake ^a^	1.22	0.34	< 0.001	1.19	0.52	0.025	1.25	0.42	0.003
Vitamin D drops	0.86	0.34	0.011	-0.20	0.83	0.811	1.13	0.33	0.001
Blood sampling season ^b^	-5.38	1.62	0.001	---	---	---	---	---	---
Parents’ birth country ^c^	-1.48	1.96	0.449	-2.46	3.26	0.451	-0.71	2.28	0.756
Household education ^d^	3.32	2.74	0.228	7.71	4.35	0.078	-0.17	3.33	0.959
Other supplements with vitamin D ^e^	2.22	1.77	0.211	-0.03	2.78	0.992	4.85	2.15	0.026

Adjusted R-squared: Full year = 0.083, summer = 0.027, winter = 0.153

^a^ Adjusted to reflect habitual consumption using the Multiple Source Method

^b^ Blood sampling season: summer (April-September) = 0, winter (October-March) = 1

^c^ Parents’ birth country: At least one parent born outside Nordics (Sweden, Norway, Denmark, Finland, Iceland) = 1, otherwise = 0

^d^ Household education: At least one parent with ≥ 12 years education = 1, otherwise = 0

^e^ Use of supplements containing vitamin D other than drops: Yes = 1, otherwise = 0

*Abbreviations: 25OHD* 25-hydroxyvitamin D, *SE* Standard error

### Main sources of vitamin D intake

**Fig. 1:**
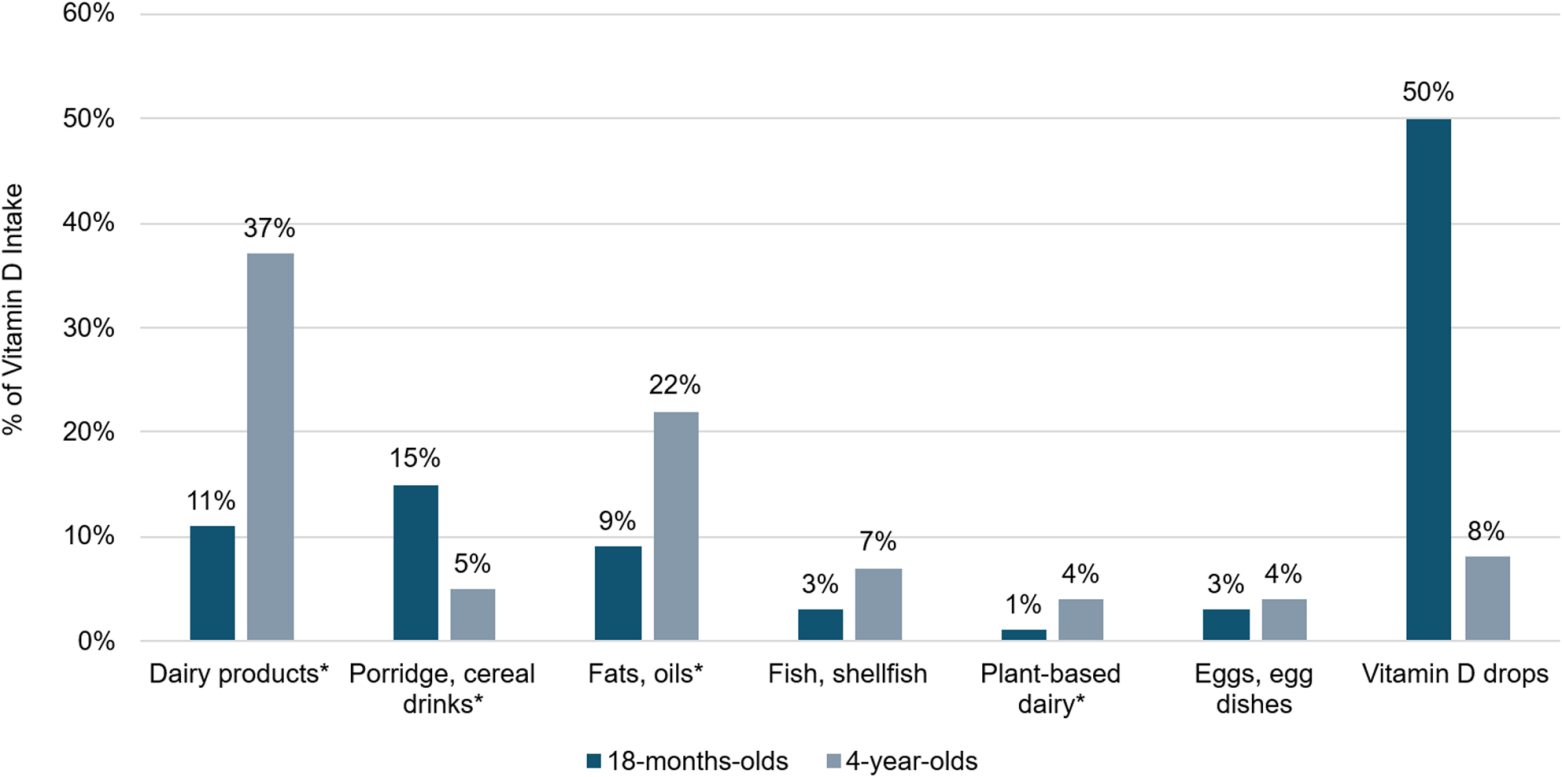
Main sources of total vitamin D intake (18-month-olds, *n* = 1074; and 4-year-olds, *n* = 746). * Denotes product categories where fortification is the predominant vitamin D source. Plant-based dairy includes alternatives to milk, yoghurt, cream and cheese

The main sources of vitamin D intake segmented by age group are presented in Fig. 1. Vitamin D drops were the largest contributor among 18mo with 50% of total vitamin D intake followed by ‘porridge and cereal drinks’ (15%) and dairy products (11%). Fortified products (excluding D drops) accounted for 38% of total vitamin D intake in this younger age group. Among 4yo, dairy products represented 37% of total vitamin D intake, followed by ‘fats and oils’ (22%). Small contributions were also observed from ‘fish and shellfish’ (7%), plant-based dairy alternatives (4%), and ‘eggs and egg dishes’ (4%). Fortified products accounted for 67% of total vitamin D intake among 4yo.

### Diet-related GHGEs segmented by age and food groups containing vitamin D

**Fig. 2:**
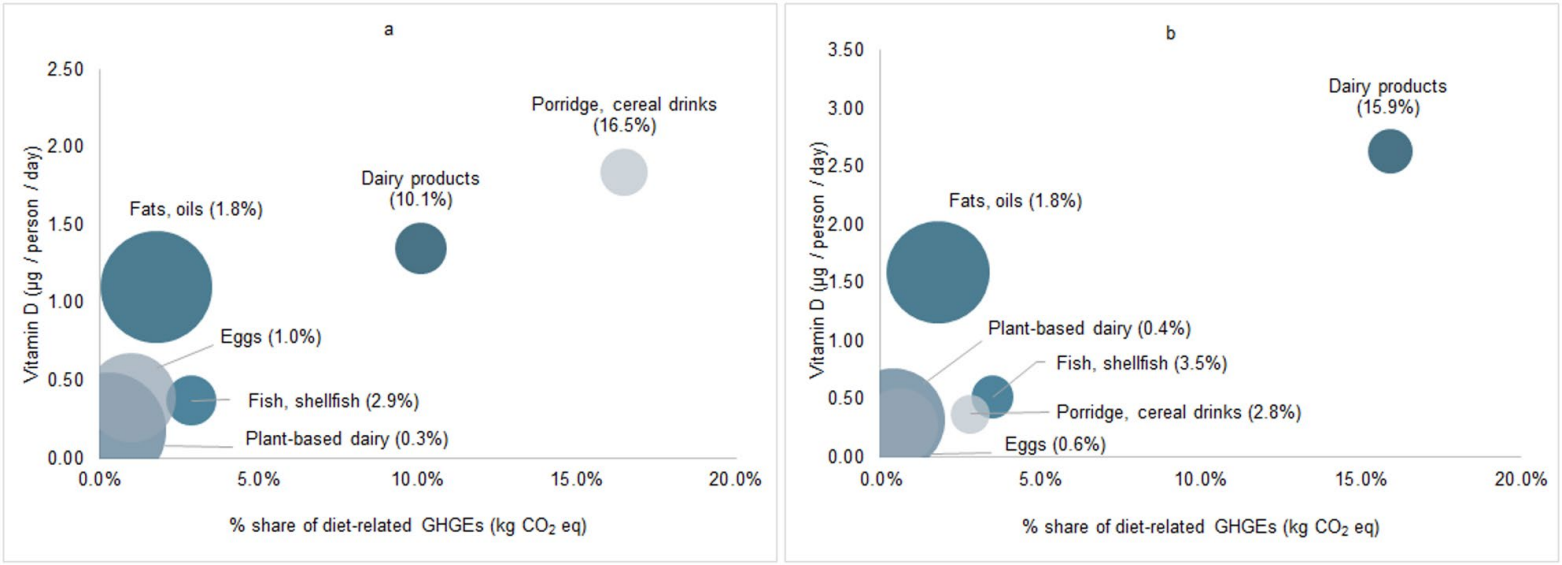
Vitamin D intake vs. share of diet-related GHGEs by food category. Vitamin D intake (y-axis) per food category in relation to % of total diet-related GHGEs measured in CO_2_ eq (x-axis), stratified by age group: (**a**) 18-month-olds, n=1074 and (**b**) 4-year-olds, n=746. Values in parentheses represent food category’s share of total diet-related GHGEs. Circle size reflects food category’s average µg vitamin D per kg CO_2_ eq. Plant-based dairy includes alternatives to milk, yoghurt, cream and cheese. Abbreviations: CO_2_ eq, CO_2_ equivalents; GHGE, greenhouse gas emissions

Total diet-related GHGEs for 18mo and 4yo were 1.7 and 1.9 kg CO_2_ eq /day, respectively. In Fig. 2, vitamin D contribution in µg/person/day per food category (y-axis) is plotted against estimated % share of total dietary GHGEs measured in CO_2_ eq (x-axis). Circle size represents categories’ average daily vitamin D contribution (µg) per kg CO_2_ eq. Among 18mo, ‘porridge and cereal drinks’ (16.5% of total GHGEs) and dairy products (10.1% of total GHGEs) had the largest climate impact among major food sources of vitamin D. For 4yo, dairy products remained the largest GHGE-contributor at 15.9% while ‘porridge and cereal drinks’ decreased to 2.8%. ‘Fats and oils’ showed a relatively low share of GHGEs in both age groups (1.8%) compared to their contribution of 1.1 µg of vitamin D for 18mo (9% of total vitamin D intake) and 1.6 µg for 4yo (22% of total vitamin D intake). More generally, plant-based dairy alternatives, ‘fats and oils’, and ‘eggs and egg dishes’ had the highest contributions of vitamin D per kg CO_2_ eq. (40–48, 37–46, and 24–25 µg/ kg CO_2_ eq, respectively) relative to other categories (porridge, cereal drinks: 6–7 µg/ kg CO_2_ eq, dairy products: 8–9 µg/ kg CO_2_ eq).

## Discussion

The national dietary survey Riksmaten Young Children 2021-24 enabled this first in-depth study of vitamin D intake and status among young children in Sweden after the 2018 expansion of mandatory fortification. The results showed a high proportion of 18-month-old (18mo) children reaching adequate vitamin D intake largely due to vitamin D drops, while 61% of 4-year-olds (4yo) did not meet the AR of 7.5 µg/d. However, no children in either age group had vitamin D deficiency (25OHD < 30 nmol/l) and only a small proportion were below 50 nmol/l. Plasma 25OHD concentrations were positively associated with vitamin D intake, from both dietary sources and vitamin D drops, and use of other supplements containing vitamin D (4yo only). 25OHD concentrations were inversely associated with blood sampling during winter (4yo only), and at least one parent born outside Nordics (18mo only). Dairy products, ‘fats and oils’, and ‘porridge and cereal drinks’ were the largest food sources of vitamin D; with 38% and 67% coming from fortified products in 18mo and 4yo, respectively. Largest contributors to diet-related greenhouse gas emissions among vitamin D-rich foods were ‘porridge and cereal drinks’ among 18mo (16% of total emissions) and dairy products among 4yo (16%).

The previous national survey of children in Sweden (Riksmaten Children 2003) included the ages 4–12 years [9]. The estimated average total vitamin D intake among 4yo in this current survey (Riksmaten Young Children 2021-24), 7.1 µg/d, is slightly higher than the 6.6 µg/d reported in 2003, perhaps reflecting effects of the expansion of Sweden’s mandatory vitamin D fortification program in 2018. Also, these intakes are still higher than similar age groups in other Nordic countries [28, 29] with the exception of Finland where vitamin D fortification is also well established with dietary intakes ranging from 8.7 to 9.6 µg/d [28]. Although the 2003 national survey did not assess vitamin D status, the Riksmaten Young Children 2021–24 study found higher levels of vitamin D sufficiency compared with earlier studies of young children in Sweden [7, 8, 11].

Our findings on the association between dietary vitamin D intake and 25OHD (β = 0.58 to1.83, depending on age group and season) are generally in line with previous cross-sectional studies on younger populations [30–34]. Randomized controlled trials with vitamin D supplementation interventions suggest slightly larger effects on 25OHD status (β = 1.55 to 2.43) but were conducted exclusively during winter, i.e., months of low sun exposure [35]. Our results also showed that vitamin D intake from both dietary sources and vitamin D drops was more strongly associated with 25OHD concentrations in 4yo during winter compared to summer, consistent with scientific evidence that dietary intake plays a greater role during periods of low sun exposure [1]. That this association was stronger in summer than in winter among 18mo (i.e., the opposite we would expect) may be partly explained by more children being sampled during early summer months when sun exposure is less intense. This may also explain why there was no association between blood sampling season and 25OHD among 18mo. This association could also be weakened by the use of vitamin D drops year-round. Use of vitamin D supplements has shown positive associations with 25OHD status in previous studies involving children [30, 33, 34, 36–38]. In our study, the association with supplement use (other than drops) was only present among 4yo during winter, again, possibly due to the enhanced role of vitamin D intake (through diet or supplements) when sun exposure is limited.

Associations between ethnicity and vitamin D status have been identified previously [34, 38], with the rationale that darker skin pigmentation and cultural-specific habits limiting sun exposure may be factors contributing to lower status. In our study, parents’ birth country was used as a proxy for a combination of factors possibly affecting vitamin D status, e.g., skin pigmentation, and influence of cultural/ethnic background on food choice and clothing practices. Among 18mo, our results showed an inverse association between vitamin D status and at least one parent being born outside the Nordics. This result could indicate that certain ethnic groups in Sweden have lower vitamin D status. Previous research has shown lower concentrations of 25OHD among adults in Sweden born outside the Nordics [39–41]. However, parents’ birth country is an imperfect proxy for ethnicity and further interpretation is limited since ethnicity is a complex, socially determined construct [42]. This variable also does not capture second-generation immigrants, which may lead to an underestimation of the diversity of participants’ ethnic backgrounds. The lack of association between parents’ birth country and vitamin D status when stratifying by blood sampling season is likely due to low statistical power from a smaller sample size combined with low proportion of parents born outside the Nordics.

The importance of vitamin D drops for 18mo achieving adequate vitamin D intake is clear. Whereas for 4yo, dairy products and ‘fats and oils’ are the main contributors to vitamin D intake. These products also contributed to vitamin D intake among 4yo in the Riksmaten Children survey in 2003 [9]. However, shares of vitamin D for both food categories have increased due to the expansion of mandatory fortification in Sweden including milk, yoghurt and margarines. From an international perspective, dairy products’ share of total vitamin D intake among 4yo in the current study (37%) is similar to that of other countries with fortification programs: Finland (40–54%), US (62%) and Canada (49%) [29].

From a climate perspective, ‘porridge and cereal drinks’ for 18mo and dairy products for 4yo, in this study, represented the highest share (16%) of diet-related GHGEs among vitamin D food sources in their respective age groups. Notably, ‘fats and oils’ contributed meaningful amounts of vitamin D in both age groups, but without high climate impacts. Although ‘fats and oils’ tended to have higher carbon footprints per gram edible amount than ‘porridge and cereal drinks’ and several dairy products, the high vitamin D fortification levels of ‘fats and oils’ (e.g., fat spreads) enabled appreciable amounts of vitamin D even at relatively low consumption. Similarly, plant-based dairy alternatives and ‘eggs, egg dishes’ were promising with substantially higher contributions of µg vitamin D per kg CO_2_ eq than ‘porridge and cereal drinks’ and dairy products. When interpreting these findings, it should be noted that climate impact from fortification was not accounted for due to lack of data. The environmental impact of fortification is often assumed to be negligible because of the small amounts added; however, this impact is currently unknown and could increase if fortification programs were expanded.

A shift toward increased consumption of plant-based dairy alternatives is of particular interest to reach adequate vitamin D intake, as these alternatives could substantially reduce dairy consumption and the associated diet-related GHGEs. Research suggests that climate sustainability of diets in Sweden is already an issue from an early age, and dairy consumption represents a large share of this burden [43]. Reliance on fortified dairy products for vitamin D intake appears to continue into adolescence [8] and may account for as high as 40–50% according to a simulation based on the expanded fortification policy in Sweden [15]. This further highlights the GHGE reduction potential of shifting from fortified dairy to plant-based dairy alternatives. Furthermore, diet modeling studies in France [44] and Australia [45] substituting meat and dairy products with plant-based alternatives have shown improvements in diet quality and minimal increases in deficiency risk.

A high reliance on fortified dairy products for vitamin D intake also raises the question of health equity. A simulation study by Forsby et al. [46] indicates that the benefit from Sweden’s expansion of vitamin D fortification has been less pronounced among pregnant women of Asian and African origin, largely due to lower consumption of fortified products, i.e., milk and yoghurt. As a result, certain population segments may be underserved by the current fortification policy. A strategy to broaden the portfolio of fortified foods would help mitigate the risk of inadequate vitamin D intake, especially among dairy non-consumers [47]. In addition, expanding mandatory fortification to foods such as bread, flour, breakfast cereals and cooking oils [48, 49] would increase flexibility to transition toward more climate-friendly diets while still achieving adequate vitamin D intake.

This study leverages an extensive dataset from a large national dietary survey of young children. However, dietary assessment methods are subject to well-known measurement errors such as participant underreporting and incorrect estimation of portion sizes [50]. It is also difficult to accurately capture intakes of foods consumed infrequently (e.g., fish and shellfish) with 2-day food records, but this issue was mitigated with the MSM statistical adjustment. Other weaknesses in the dataset include a skewness towards children born in Sweden, and families originating from the Nordics with higher education levels and higher disposable income [17]. This limits the external validity of our results, as findings may not be generalizable to more diverse or socioeconomically varied populations. Also, vitamin D intake from supplements other than vitamin D drops could not be estimated. A lack of variables related to sun exposure (e.g., UVB light exposure, sun protection, clothing, and travel to sunny locations) also prevented more detailed analysis on determinants of vitamin D status. More targeted variables capturing physiological factors such as skin pigmentation and genetic factors related to vitamin D metabolism would also have been desirable.

The RISE Food and Climate Database was an invaluable asset to this study, an extensive database with foods most relevant for the Swedish market and climate data representative of Swedish food consumption. Limitations here include LCA data representing only up to factory gate, causing possible under-estimation of total climate impact, and food groupings potentially hiding varying climate impacts of individual foods. For example, more detailed dietary data may be required for categories like ‘fish and shellfish’ (e.g., species, production methods) to provide more specific guidance on less climate intensive food choices [51]. The lack of LCA data on the climate impact of fortification is an additional limitation but reflects common practice in current research. Further studies are needed to assess the environmental impact of dietary shifts toward fortified products. Finally, environmental metrics such as land use, water use, biodiversity impact and pesticide use were not considered to be within the scope of this study but have been used elsewhere [43].

## Conclusions

We conclude that a high proportion of young children in Sweden have sufficient vitamin D status with major determinants including vitamin D intake (both from the diet and vitamin D drops) and season of blood sampling. Vitamin D drops are a key source of vitamin D for 18-month-olds, whereas 4-year-olds rely on fortified dairy, a major contributor to diet-related GHGEs. Dependence on fortified dairy as a primary dietary source of vitamin D raises climate concerns, which highlights that a shift towards vitamin D fortified plant-based alternatives could substantially reduce the climate impact from these products and represents an important direction for future research.

## Supplementary Information

Additional file 1.

## Data Availability

Data used for the analyses in this study are not publicly available to preserve individuals’ privacy under the European General Data Protection Regulation.

## Abbreviations

18mo: 18-month-olds
4yo: 4-year-olds
25OHD: 25-hydroxyvitamin D
AR: Average requirement
CO_2_ eq: Carbon dioxide equivalents
GHGE: Greenhouse gas emission
LCA: Life cycle assessment
MSM: Multiple source method
