# Supplementary material for: High proportion of young children are vitamin D sufficient after expansion of Sweden’s mandatory fortification but dairy products also contribute to a high climate impact
Source: Nutr J. 2026 Apr 1;25:41. doi: 10.1186/s12937-026-01318-6 (PMC13064400; doi:10.1186/s12937-026-01318-6)
Supplement: Supplementary file 1 — Additional file 1. [file 12937_2026_1318_MOESM1_ESM.pdf]

Supplementary materials

Table S1 Food categories used in the national dietary survey Riksmaten Young Children 2021-24 (translated from Swedish).

Category names	Example foods
Baby food	Infant formula, baby food jars and pouches
Bread	White bread, wholegrain, hotdog buns
Breakfast cereals	Breakfast cereals, granola, müsli
Candy and chocolate	Candy, milk chocolate, cookies
Cereal grains	Bulgur, wheat, oats, couscous
Cheese and cheese dishes	Hard cheese, cottage cheese, quark
Dairy products	Milk, breast milk, yoghurt, smoothies
Drinks	Water
Egg dishes	Boiled eggs, scrambled eggs, omelett
Fats and oils	Fat spreads, butter, margarine, vegetable oils
Fish and shellfish	Fish sticks, salmon, white fish
Fruits and berries	Bananas, apples, pears, oranges
Fruit juice	Apple juice, orange juice
Ice cream	Popsicles, ice cream
Meat dishes	Meatballs, pasta meat sauce, taco meat, chili, beef stew
Nuts and seeds	Peanut butter, peanuts, cashews
Offal, organ meets	Blood sausage, liver
Other	Spices, broth (vegetable, chicken, beef)
Other sweet foods	Fruit preserves, apple sauce
Pancakes, etc.	Pancakes, waffles, crepes
Pasta dishes	Pasta (boiled), lasagna, macaroni and cheese
Pastries, desserts and crackers	Cookies, cakes, buns, muffins, pies

Pizza, hamburgers, etc.	Pizza, sandwiches, hamburgers
Plant-based dairy alternatives	Oat drink, plant-based yoghurts, cream, and cheese
Plant-based meat alternatives	Tofu, vegetarian dishes
Porridge and cereal drinks	Porridge of oats, rice, etc. and children's porridge and cereal drinks (*välling*)
Potato dishes	Boiled potatoes, mashed potatoes, french fries
Poultry dishes	Grilled chicken, chicken stew, chicken nuggets
Rice dishes and rice cakes	Boiled rice, rice noodles
Sausage dishes and coldcuts	Hot dogs, sausages, sandwich meats (red meat, pork)
Snacks	Popcorn, chips
Soft drinks and fruit drinks	Fruit-flavored drinks, soda
Soup and salad	Soup, tacos, salad
Spread and sauces	Ketchup, pasta sauce, mayonnaise, bernaise
Vegetables and vegetable dishes	Tomatoes, green peas, cucumbers, carrots, corn, broccoli

Figure S1 Directed acyclic graph for selection of determinants and confounders relevant to blood concentrations of 25-hydroxyvitamin D

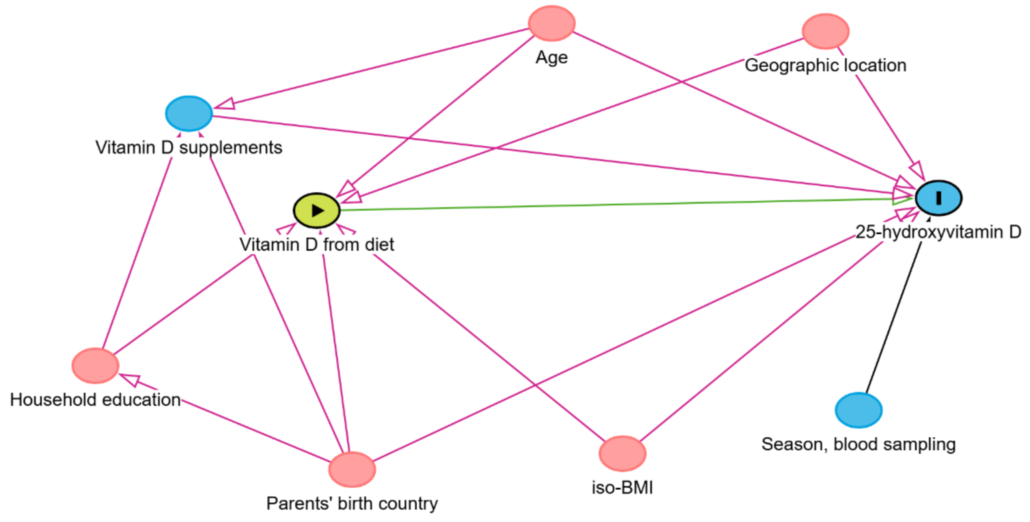

Table S2 Parents' birth countries grouped by region, all participants excluding Sweden, Norway, Denmark, Finland and Iceland (n=341)

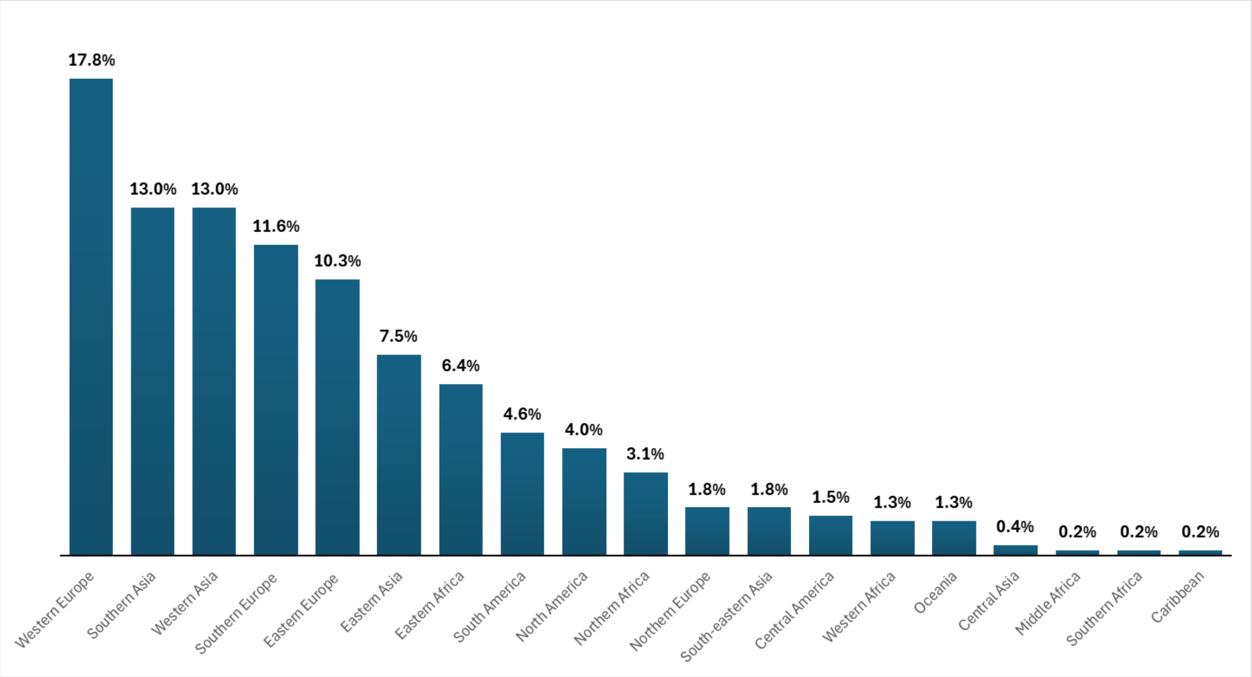

Regional groupings per the United Nations' geographic regions for statistical use (https://unstats.un.org/unsd/methodology/m49/)

Table S3 Vitamin D intake and status stratified by month of blood sampling

Month of sampling	Jan - Mar	Apr - Jun	Jul - Sep	Oct - Dec
18-month-olds:				
Participants, n (%)	111 (40)	97 (35)	1 (0)	72 (25)
Mean vitamin D intake, µg /d *	12.9	12.6	13.8	12.7
Mean total 25OHD, nmol/l	71.8	75.2	66	72.5
% below 50 nmol/l	9.9	3.1	0.0	8.3
4-year-olds:				
Participants, n (%)	68 (25)	81 (30)	60 (22)	61 (23)
Mean vitamin D intake, µg /d *	7.8	6.9	6.4	7.8
Mean total 25OHD, nmol/L	67.8	68.3	75.2	66.6
% below 50 nmol/l	1.5	7.4	0.0	8.2

* Individual vitamin D intakes adjusted by Multiple Source Method to reflect habitual intake.
Abbreviations: 25OHD, 25-hydroxyvitamin D

Article title: High proportion of young children are vitamin D sufficient post-expansion of Sweden's mandatory fortification but dairy products also contribute to a high climate impact

Authors: André Hesselink, Anna Winkvist, Anna Karin Lindroos, Lotta Moraeus, Helena Bjermo, Sanna Lignell, Linnea Bärebring, Elinor Hallström, Hanna Augustin

Corresponding author:

André Hesselink
Sahlgrenska Academy, University of Gothenburg
andre.hesselink@gu.se
